# Glyphosate treatment mediates the accumulation of small discrete 5′- and 3′-terminal fragments of 18S rRNA in plant cells

**DOI:** 10.18699/VJGB-23-13

**Published:** 2023-04

**Authors:** A.V. Zhigailov, A.S. Nizkorodova, K.O. Sharipov, N.S. Polimbetova, B.K. Iskakov

**Affiliations:** M.A. Aitkhozhin Institute of Molecular Biology and Biochemistry of the Ministry of Science and Higher Education of the Republic of Kazakhstan, Almaty, Kazakhstan; M.A. Aitkhozhin Institute of Molecular Biology and Biochemistry of the Ministry of Science and Higher Education of the Republic of Kazakhstan, Almaty, Kazakhstan; M.A. Aitkhozhin Institute of Molecular Biology and Biochemistry of the Ministry of Science and Higher Education of the Republic of Kazakhstan, Almaty, Kazakhstan; M.A. Aitkhozhin Institute of Molecular Biology and Biochemistry of the Ministry of Science and Higher Education of the Republic of Kazakhstan, Almaty, Kazakhstan; M.A. Aitkhozhin Institute of Molecular Biology and Biochemistry of the Ministry of Science and Higher Education of the Republic of Kazakhstan, Almaty, Kazakhstan

**Keywords:** wheat embryos, 18S rRNA, discrete fragmentation, 40S ribosomal subunits, glyphosate, eIF2α phosphorylation, stress, starvation, зародыши пшеницы, 18S рРНК, дискретная фрагментация, 40S рибосомные субъединицы, глифосат, фосфорилирование eIF2α, стресс, голодание

## Abstract

Under many kinds of stress, eukaryotic cells rapidly decrease the overall translation level of the majority of mRNAs. However, some molecular mechanisms of protein synthesis inhibition like phosphorylation of eukaryotic elongation factor 2 (eEF2), which are known to be functional in animals and yeast, are not implemented in plants. We suggest that there is an alternative mechanism for the inhibition of protein synthesis in plant cells and possibly, in other eukaryotes, which is based on the discrete fragmentation of 18S rRNA molecules within small ribosomal subunits. We identified four stress-induced small RNAs, which are 5’- and 3’-terminal fragments of 18S rRNA. In the present work, we studied the induction of 18S rRNA discrete fragmentation and phosphorylation of the α-subunit of eukaryotic initiation factor 2 (eIF2α) in germinated wheat embryos in the presence of glyphosate, which imitates the condition of amino acid starvation. Using northern and western blotting, we have shown that stress-induced 18S rRNA fragments started to accumulate in wheat embryos at glyphosate concentrations that did not evoke eIF2α phosphorylation. It was also found that cleavage of 18S rRNA near the 5’-terminus began much earlier than eIF2α phosphorylation, which became noticeable only at higher concentration (500 μM) of glyphosate. This result suggests that discrete fragmentation of 18S rRNA may constitute a regulatory mechanism of mRNA translation in response to stress and may occur in plant cells in parallel with and independently of eIF2α phosphorylation. The identified small 5’- and 3’-terminal fragments of 18S rRNA that accumulate during various stresses may serve as stress resistance markers in the breeding of economically important plant crops.

## Abstract (ru)

При многих видах стресса эукариотические клетки быстро снижают общий уровень трансляции большинства мРНК. Однако некоторые молекулярные механизмы ингибирования синтеза белка, такие как фосфорилирование эукариотического фактора элонгации трансляции (eEF2), функционируют у животных и дрожжей, но не реализуются у растений. Мы предполагаем, что существует альтернативный механизм ингибирования синтеза белка в клетках растений и, возможно, других эукариот, основанный на дискретной фрагментации молекул 18S рРНК внутри малых субъединиц рибосомы. Мы идентифицировали четыре малые РНК, индуцированные стрессом, которые представляют собой 5’- и 3’-концевые фрагменты 18S рРНК. В настоящей работе мы исследовали индукцию дискретной фрагментации 18S рРНК и фосфорилирование α-субъединицы эукариотического фактора инициации 2 (eIF2α) в проросших зародышах пшеницы в присутствии глифосата, имитирующего состояние аминокислотного голодания. Используя нозерн- и вестерн-блоттинг, мы показали, что индуцированные стрессом фрагменты 18S рРНК начинают накапливаться в зародышах пшеницы при концентрациях глифосата, не вызывающих фосфорилирования eIF2α. Также установлено, что расщепление 18S рРНК вблизи 5’-конца начинается гораздо раньше, чем становится заметным фосфорилирование eIF2α при высокой концентрации глифосата (500 мкМ). Этот результат указывает на то, что дискретная фрагментация 18S рРНК может представлять собой регуляторный механизм трансляции мРНК в ответ на стресс и происходить в растительных клетках параллельно с фосфорилированием eIF2α и независимо от него. Выявленные 5’- и 3’-концевые малые фрагменты 18S рРНК, накапливающиеся при различных стрессах, могут служить маркерами стрессоустойчивости в процессе селекции хозяйственно важных культур растений.

## Introduction

Protein biosynthesis is a very energy-intensive process, so under stress conditions, the translation of most cellular mRNAs is inhibited in order to save energy and resources and to ensure preferential synthesis of stress proteins. Several molecular mechanisms of protein synthesis inhibition have been described in mammalian and yeast cells. One of these mechanisms is the eukaryotic translation elongation factor 2 (eEF2) phosphorylation, which is carried out by a highly specific protein kinase in response to a sharp decrease in cytosolic ATP concentration levels. Phosphorylation inactivates mammalian eEF2 by preventing it from binding to the ribosome (Ballard et al., 2021). However, plants do not exhibit endogenous kinase activity for eEF2 either under normal conditions (Smailov et al., 1993), or under stress (Gallie et al., 1998).

The second mechanism known in animals to reduce the level of mRNA translation is triggered under conditions of amino acid starvation and is mediated by eIF4E-binding proteins (4E-BPs), which prevent eIF4E from binding to the m7Gcap structure of mRNA (Hernandez et al., 2010). However, no clear homologs of these eIF4E-BPs have yet been found in plants (Echevarria-Zomeno et al., 2013), nor were any orthologues of the 4E-BPs genes found in plant genomes (Browning, Bailey-Serres, 2015).

Another important mechanism of eukaryotic protein synthesis inhibition is the phosphorylation of the α-subunit of eukaryotic initiation factor 2 (eIF2α) by specific protein kinases. This process in mammalian and yeast cells leads to the blocking of GDP → GTP exchange protein eIF2B and to a sharp inhibition of the mRNA translation initiation (Baird, Wek, 2012). However, recycling of the ternary complex in plant cells can occur without the participation of eIF2B (Shaikhin et al., 1992), and eIF2α phosphorylation in plant systems in vitro does not lead to strong inhibition of protein synthesis (Zhigailov et al., 2020). In addition, of the four protein kinases (mPKR, mHCR, mPERK, mGCN2) that phosphorylate the eIF2α in mammalian cells, only pGCN2-kinase was found in plants, and the phosphorylation of eIF2α in plants is not a universal response to all stress types (Immanuel et al., 2012; Zhigailov et al., 2020).

Thus, the mechanisms of protein biosynthesis suppression due to the phosphorylation of translational factors, which are well described for mammals and yeast, are either used to a limited extent or are not realized at all in plant cells. We suggest that another mechanism of protein synthesis inhibition can function in plants, which is triggered by certain abiotic and biotic stresses. In our understanding, this mechanism is associated with the cleavage at certain sites of the 18S rRNA as part of 40S ribosomal subunits (40S RS). Previously, we described the process of 18S rRNA cleavage, leading to 5′-terminal fragments formation of 132–134 nt. (Zhanybekova et al., 1996) and of 54–57 nt. (Zhigailov et al., 2014), as well as a 3′-terminal fragment of 100 nt. (Zhigailov et al., 2013). Our data are quite consistent with the data of full-transcriptome analysis, which showed that breaks in 28S-, 18S-, and 5.8S-rRNA do not occur randomly, but discretely, which leads to the fact that some fragments of ribosomal RNA are detected in the cell significantly more often than other fragments (Chen et al., 2017).

The process of RNA cleavage is widely used by cells during the processing of ribosomal RNA from their precursor during ribosome biogenesis (Henras et al., 2015). In addition, in proand eukaryotic organisms, the mechanism of protein biosynthesis suppression is realized due to the cleavage of the 28S rRNA molecule from the large (60S) ribosomal subunit along the sarcin-ricin loop with the cleavage of the 3′-terminal EndorRNA- fragment (Endo, 1988). Toxins of plants (ricin, abrin, and modecin), fungi (α-sarcin) and bacteria (Shiga toxin) act this way (Kast et al., 2014). Possibly similar endonucleases and/or glycosylases (that mediate abasic site formation as in the case of ribosome inactivating proteins, RIPs) are activated in plant cells during stress, but targeting 18S rRNA in 40S RS instead of 28S rRNA in 60S RS, and thus leading to temporary or permanent suppression of mRNA translation.

In this work, we have shown that in the case of glyphosatemediated amino acid starvation, when the only specific eIF2α kinase of plants (pGCN2-kinase) is activated, in addition to plant eIF2α phosphorylation, another protective mechanism is triggered in plant cells, namely, discrete fragmentation of 18S rRNA. It was shown that the accumulation in plants of 18S rRNA 5′-terminal fragments of 75 nucleotides (75nt- 5′18S) and 134 nucleotides (134nt-5′18S) begins earlier than the activation of pGCN2 kinase and becomes noticeable at relatively low concentrations of glyphosate when plant eIF2α phosphorylation does not occur at all.

## Materials and methods

Plant material and treatment. Wheat (Triticum aestivum L. cv. Kazakhstanskaya 10) seeds were sterilized in 70 % (v/v) ethanol for 2 min, then in 2 % (w/v) NaOCl for 20 min, and washed thoroughly with sterile water. Seeds were germinated at 26 ºС on sterile filter paper soaked in water. After 18 hours, viable embryos were isolated by spatula from swollen seeds and placed in 1 % glucose solution containing 50 U/ml penicillin, 50 μg/ml chloramphenicol, and 50 μg/ml nystatin. After this, embryos were divided into equal portions (1 g), which were subjected to treatment with glyphosate (simulation of amino acid starvation) or without any additives (control).

Synthesis of probes. DIG-labeling of de novo synthetized oligodeoxyribonucleotides 5′18S (5′-ACAAGCATATGA CTACTGGCAGGATCAACCAGGTA) and 3′18S (5′-CAA TGATCCTTCCGCAGGTTCACCTACGGAAACCT) was carried out using DIG Oligonucleotide 3′-End Labeling Kit (Roche) according to the manufacturer’s manual. Probes (5′18S-DIG and 3′18S-DIG) were used for northern blotting

Northern blotting. Total RNA was extracted from plant tissues with Tri-reagent (Sigma Aldridge) and analyzed on 10 % PAGE with 8 M urea in Tris-borate buffer (1xTBE: 89 mM Tris-borate, 2 mM EDTA, pH 8.3). RNAs were blotted to a nylon membrane (Roche) equilibrated in 0.1x TBE using a semi-dry blotter (Sci-Plas) at 250 mA for 30 min. The membrane was dried and irradiated with UV light for 2 min at 10 mJ/cm2 in a crosslinker (UVP). Hybridization of DIG-labeled probes and subsequent chemiluminescent band detection was performed with DIG Luminescent Detection Kit for Nucleic Acids (Roche) according to the manufacturer’s procedure. The hybridization temperature was 55 °C. Anti-Digoxigenin-AP Fab fragment conjugates (Roche) were used to detect bound DIG-labeled probes. The blots were developed using a commercial alkaline phosphatase substrate CSPD (Roche).

SDS-polyacrylamide gel electrophoresis (SDS-PAGE) and immunoblotting. Frozen embryos were ground to a powder in a mortar and then homogenized in Laemmli sample buffer (Laemmli, 1970). Proteins were separated by 12.5 % SDS-PAGE with 0.1 % SDS. The separated proteins were transferred to a nitrocellulose membrane (GVS) that was afterwards stained with Ponceau S (Sigma-Aldrich). The antibodies against human phospho-eIF2α (S51) produced in rabbit (CellSignaling Technology, 1:1000) were used for the immune-detection of phosphorylated T. aestivum (Ta) eIF2α (TaeIF2(αP)). Then horseradish peroxidase-conjugated antirabbit secondary antibodies produced in donkey (ECL, 1:2000 dilution) were used.

## Results

**Fig. 1.**
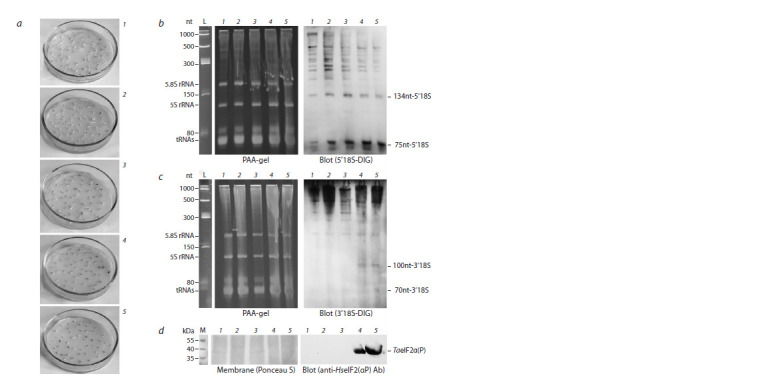
The effect of glyphosate on TaeIF2α phosphorylation and 18S rRNA fragmentation in germinated wheat embryos. a, The appearance of wheat embryos exposed to different concentration of glyphosate; b, Northern blot analysis using 5’18S-DIG probe (right panel). Left panel – ethidium bromide stained PAA-gel; c, Northern blot analysis using 3’18S- DIG probe (right panel). Left panel – ethidium bromide stained PAA-gel; d, Phosphorylation status of TaeIF2α in wheat embryos. Presented are the membrane stained with Ponceau S (left panel) and blot-membrane developed using anti-HseIF2(αP) antibodies (right panel). For all variants, embryos were first germinated at 26 °C for 18 h and then incubated at 26 °C for 10 h in the absence or presence of glyphosate at the following concentration: 1 – 0 μM (control); 2 – 5 μM; 3 – 50 μM; 4 – 0.5 μM; 5 – 5 μM. L – Low Range ssRNA ladder; M – PageRuler Plus Protein Ladder.

The effect of glyphosate concentration on 18S rRNA fragmentation in wheat embryos. Since the mechanisms of mRNA translation inhibition mediated by 4E-BPs and eEF2K are not implemented in plants, it is believed that the main response in plants to amino acid starvation is eIF2α phosphorylation with pGCN2 kinase (Zhang et al., 2008). To test whether the process of discrete fragmentation of 18S rRNA is also induced under these conditions, the herbicide glyphosate was used. Glyphosate targets 5-enolpyruvoylshikimate 3-phosphate synthase, which catalyzes the key penultimate reaction in the shikimate pathway (Padgette et al., 1995). Therefore, it inhibits the synthesis of many aromatic plant metabolites including the amino acids tryptophan, tyrosine, and phenylalanine and leads to pGCN2 kinase activation and phosphorylation of the plant eIF2α (Zhang et al., 2008). Germinated wheat embryos were treated with glyphosate at various concentrations, after which the content of 18S rRNA small fragments and the phosphorylation status of TaeIF2α were assessed in their cells. The results are present in Figure 1.

Phosphorylation of TaeIF2α becomes noticeable only at relatively high concentrations (0.5 and 5 μM) of glyphosate (tracks 4 and 5 on Fig. 1, d; right panel), at which wheat embryos stopped to grow (variants 4 and 5 on Fig. 1, a). The appearance of 3′-terminal fragments 100nt-3′18S and 70nt- 3′18S was observed at the same concentrations of glyphosate (tracks 4 and 5 on Fig. 1, c; right panel). At the same time, 5′-terminal fragments of 18S rRNA, 134nt-5′18S and 75nt- 5′18S, began to accumulate in noticeable amounts even at very low concentrations (5 μM) of glyphosate (see Fig. 1, b). The results of semi-quantitative optical densitometry analysis for this experiment are presented in Table 1. Since the 134nt- 5′18S fragment can be a precursor of 75nt-5′18S, and the 100nt-3′18S fragment can act as a precursor for 70nt- 3′18S, it is reasonable to estimate the sum of these small 18S rRNA fragments.

**Table 1.**
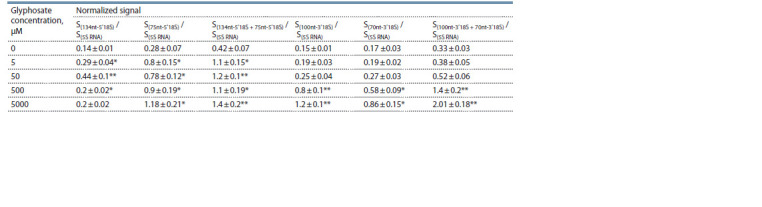
Optical densitometry analysis for assessing the 18S rRNA fragments in wheat embryos following treatment with glyphosate at various concentrations

**Fig. 2.**
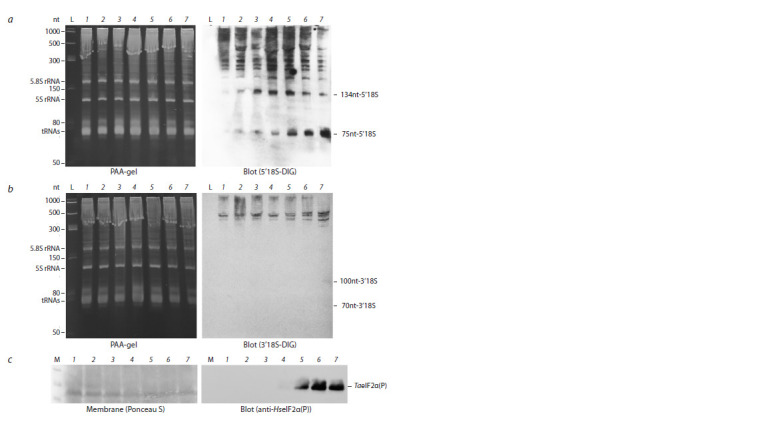
The dynamics of glyphosate action on the TaeIF2α phosphorylation and 18S rRNA fragmentation in germinated wheat embryos. a, Northern blotting analysis (right panel) using 5’18S-DIG probe. Left panel – ethidium bromide stained PAA-gel; b, Northern blotting analysis (right panel) using 3’18S-DIG probe. Left panel – ethidium bromide stained PAA-gel; c, Phosphorylation status of TaeIF2α in wheat embryos exposed to glyphosate treatment. Presented are the membrane stained with Ponceau S (left panel) and blot-membrane developed using anti-HseIF2(αP) antibodies (right panel). The embryos were first germinated at 26 °C for 18 h and then incubated at 26 °C in the presence of 0.5 μM glyphosate during the following periods: 1 – 0 min; 2 – 15 min; 3 – 30 min; 4 – 45 min; 5 – 60 min; 6 – 90 min; 7 – 180 min. L – Low Range ssRNA ladder; M – PageRuler Plus Protein Ladder.

The dynamics of glyphosate influence on 18S rRNA fragmentation in wheat embryos. Then, we assessed how quickly wheat embryos respond to glyphosate treatment by measuring the time dependence of TaeIF2α phosphorylation and of discrete fragmentation of 18S rRNA. For this, a glyphosate concentration of 500 μM was chosen, which induced quite effective phosphorylation of TaeIF2α, as well as a significant increase in the content of 18S rRNA small fragments: 134nt- 5′18S, 75nt-5′18S, 100nt-3′18S and 70nt-3′18S (see Fig. 1, Table 1). The results of the experiment are shown in Figure 2.

The results of semi-quantitative optical densitometry analysis of the data presented in Figure 2 are shown in Table 2.

**Table 2.**
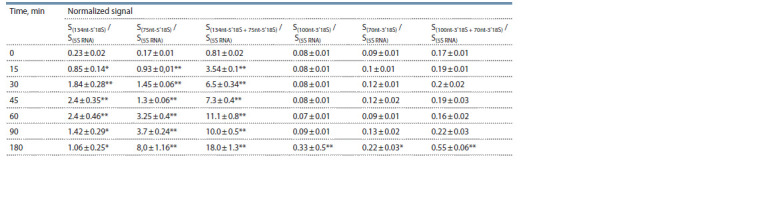
Densitometry analysis for assessing the 18S rRNA fragments in wheat embryos treated with 500 μM glyphosate for different time periods

Data presented in Figure 2 and Table 2 show that TaeIF2α phosphorylation begins 45 min after the start of glyphosate treatment (a faintly visible band on track 4 on Fig. 2, c; right panel), and TaeIF2αP becomes quite noticeable after 60 min of such treatment (track 5 in Fig. 2, c; immunoblot).

The 3′-terminal fragmentation of 18S rRNA is observed after 3 hours after the start of glyphosate treatment: fragments 100nt-3′18S and 70nt-3′18S become detectable as faintly visible bands on track 7 of Figure 2, b (right panel). The amount of these 3′-coterminal fragments is significantly lower than after 10 hours of the same treatment with glyphosate (compare with track 4 on Fig. 1, c; right panel).

As for fragmentation from the 5′-terminus of 18S rRNA, the amount of both 5′-coterminal fragments, 134nt-5′18S and 75nt-5′18S, is significantly increased as early as by the 15th min after the start of treatment of wheat embryos with glyphosate (see Fig. 2, a; right panel). Notably, the amount of the fragment 134nt-5′18S is higher than that of 75nt-5′18S fragment during 30–45 min of incubation with glyphosate. By 60 min of incubation their amounts become almost equal and after that, the amount of 75nt-5′18S fragment becomes higher (by 90 min) and even obviously prevalent by 180 min (see Fig. 2, a; right panel). Similar interrelation can be seen in Figure 1, b (right panel) regarding the applied concentrations of glyphosate.

These observations suggest that cleavage at 134th nucleotide may happen more quickly and this site is more susceptible at the beginning of stress. The cleavage site at 75th nucleotide becomes more prevalent with an increase of stress duration and severity. The cleavage sites at the 3′-terminal segment of 18S rRNA occur only at very high severity and duration of stress. Therefore, there seemingly exist several different mechanisms for the cleavage at 5′- and 3′-termini of 18S rRNA, which may result in several different consequences for the functioning of 40S RS.

## Discussion

No phosphorylation of eIF2α was observed in plants under osmotic and oxidative stresses (Lageix et al., 2008), heat shock (Gallie et al., 1997; Echevarria-Zomeno et al., 2013) and during unfolded protein response in plants (Kamauchi et al., 2005). At the same time, during these stresses a significant decrease in the translation level of most mRNAs is observed with exception only for those templates that are responsible for the synthesis of stress proteins (Altschuler, Mascarenhas, 1982; Ruberti et al., 2015). Most likely, in plants, other mechanisms of protein biosynthesis suppression are realized, than eIF2α phosphorylation (Yu et al., 2021). In addition, eIF2α phosphorylation is not the only possible mechanism of response to some types of stress in different eukaryotic cells. For example, when yeast cells are exposed to harsh ultraviolet light, phosphorylation of eIF2α is observed, as well as a suppression of the overall level of protein synthesis. However, inhibition of mRNA translation upon exposure to UV light occurs even in cells containing a mutant form of eIF2α that is not capable of phosphorylation (Knutsen et al., 2015).

We postulate that the process of discrete fragmentation of 18S rRNA observed under glyphosate mediated amino acid starvation may lead to a decrease in the level of mRNA translation. This molecular mechanism can be realized in parallel with the known mechanism of translational regulation mediated by eIF2α phosphorylation and independently of it.

Understanding the molecular mechanisms of plant adaptation to stresses can make it possible to increase the efficiency of breeding work to obtain genetic lines and varieties of economically important plant species that are characterized by increased resistance to certain stresses.

## Conclusion

This paper presents data indicating that in plant cells the imitation of amino acid starvation induces, in addition to eIF2α phosphorylation, another cellular response that involves the cleavage of the 18S rRNA molecule with the formation of discrete 5′- and 3′-terminal fragments. At the same time, 3′-terminal fragments of 18S rRNA appear only at lethal concentrations of glyphosate and after a prolonged period of stress (3 hours or more). In contrast, 5′-terminal fragments of 18S rRNA began to accumulate in wheat embryos at relatively low glyphosate concentrations, at which wheat embryos could continue development, and already 15 min after the start of glyphosate treatment. Thus, the process of 18S rRNA fragmentation in wheat embryo 40S RS is triggered even under conditions where eIF2α phosphorylation does not occur. We suggest that such cleavage of the 18S rRNA molecule, which is activated during amino acid starvation, may result in either global or selective suppression of mRNA translation.

## Conflict of interest

The authors declare no conflict of interest.
